# Fiber Specific Changes in Sphingolipid Metabolism in Skeletal Muscles of Hyperthyroid Rats

**DOI:** 10.1007/s11745-013-3769-3

**Published:** 2013-03-07

**Authors:** A. Chabowski, M. Żendzian-Piotrowska, A. Mikłosz, B. Łukaszuk, K. Kurek, J. Górski

**Affiliations:** Department of Physiology, Medical University of Bialystok, ul. Mickiewicza 2C, 15-222 Bialystok, Poland

**Keywords:** Ceramide, Sphingolipids, Triiodothyronine, Skeletal muscle

## Abstract

Thyroid hormones (T_3_, T_4_) are well known modulators of different cellular signals including the sphingomyelin pathway. However, studies regarding downstream effects of T_3_ on sphingolipid metabolism in skeletal muscle are scarce. In the present work we sought to investigate the effects of hyperthyroidism on the activity of the key enzymes of ceramide metabolism as well as the content of fundamental sphingolipids. Based on fiber/metabolic differences, we chose three different skeletal muscles, with diverse fiber compositions: soleus (slow-twitch oxidative), red (fast-twitch oxidative-glycolytic) and white (fast-twitch glycolytic) section of gastrocnemius. We demonstrated that T_3_ induced accumulation of sphinganine, ceramide, sphingosine, as well as sphingomyelin, mostly in soleus and in red, but not white section of gastrocnemius. Concomitantly, the activity of serine palmitoyltransferase and acid/neutral ceramidase was increased in more oxidative muscles. In conclusion, hyperthyroidism induced fiber specific changes in the content of sphingolipids that were relatively more related to de novo synthesis of ceramide rather than to its generation via hydrolysis of sphingomyelin.

## Introduction

Thyroid hormones (T_3_, T_4_) are well known modulators of whole body energy utilization. However, they also serve as an important molecular signaling transducers [1]. A number of cellular signaling pathways are modified by triiodothyronine (T_3_), including the sphingomyelin/ceramide pathway [2]. Skeletal muscles constitute the bulk of the body’s total metabolic activity either by virtue of its mass or oxidative capacity, and thus represent an important target for the action of T_3_ [3]. Furthermore, others indicate high expression of T_3_ receptor (THR) in muscle fibers [4] and interestingly, the thyroid hormone receptors density is muscle specific as slow-twitch (oxidative) fibers are more sensitive to T_3_ than fast-twitch (glycolytic) muscles [5].

Recently, diminished content of ceramide with a concomitant increase in sphingomyelin concentration, in skeletal muscles was reported in hypothyroid rats [6]. Ceramide content in myocytes is a net result of myocellular ceramide generation (by hydrolysis of sphingomyelin or de novo synthesis pathway) and its degradation (by ceramidases) [7]. The formation of ceramide de novo begins with condensation of serine and palmitoyl-CoA, the reaction catalyzed by serine palmitoyltransferase (SPT). However, hydrolysis of sphingomyelin, can also generate substantial amounts of ceramide, through the increased activity of specific sphingomyelinases (acidic and/or neutral sphingomyelinases—aSM-ase/nSM-ase). In contrast, degradation of ceramide occurs mainly by its deacylation and formation of sphingosine in a reaction catalyzed by specific ceramidases (CDase, acidic, neutral and alkaline). Sphingosine might also be further phosphorylated to sphingosine-1 phosphate. In addition, it should be noted that most of the steps involved in sphingolipid metabolism is reversible [8]. Increasing evidence strongly supports involvement of different sphingolipids in the regulation of myocellular functions [9, 10], and disturbances in sphingolipid formation have been found essential in the induction of pathologies [2]. More specifically, elevated concentrations of ceramide or other sphingolipid intermediates are implicated in the induction of insulin resistance in skeletal muscle [8]. However, after exercise (a known insulin-sensitizing effect), higher ceramide content was also reported in muscles, in animals [11] and humans [12].

At present, it is unclear whether thyroid hormones influence sphingolipid metabolism in skeletal muscles. Therefore, the activity of key enzymes of ceramide metabolism (serine palmitoyltransferase, neutral sphingomyelinase, acid sphingomyelinase, neutral ceramidase and alkaline ceramidase) as well as the content of sphingolipid metabolism products (sphingosine, sphinganine, sphingosine-1-phosphate, ceramide and sphingomyelin) were measured. To investigate the effects of hyperthyroidism, rats were made hyperthyroid (*n* = 8) over 10 days using T_3_ i.p. injections and subsequently three types of skeletal muscles were taken: soleus (slow-twitch oxidative), red (fast-twitch oxidative-glycolytic) and white (fast-twitch glycolytic) section of gastrocnemius.

## Materials and Methods

The experimental protocol was approved by the Ethical Committee for Animal Experiments at the Medical University of Białystok. Male Wistar rats (200–220 g body weight) were housed under controlled conditions (21 °C ± 2, 12 h light/12 h dark cycle) with unlimited access to standard chow and water. The animals were divided into two groups, control (*n* = 8) and treated with triiodothyronine (T_3_) (*n* = 8). Triiodothyronine (Sigma–Aldrich, St. Louis, MO) was injected subcutaneously with a dose of 100 μg/100 g of body weight, daily for 10 days [13]. This dose is quite commonly used to mimic hyperthyroidism in humans [13, 14]. Control rats were treated with saline accordingly. After 10 days, rats were fasted overnight and anaesthetized by intraperitoneal injection of pentobarbital with a dose of 80 mg/kg of body weight. Fasting blood samples were collected sodium-heparinized tubes and centrifuged at 20,000×*g* for 20 min at 4 °C. Plasma was removed and stored at −80 °C until analyzed. Concomitantly, the soleus, red and white section of gastrocnemius were excised, cleaned of blood and/or connective tissue and immediately frozen in liquid nitrogen and then stored at −80 °C until analysis.

### Sphingomyelin Content

The tissue samples were pulverized in an aluminum mortar precooled previously in liquid nitrogen. The powder was then transferred to a tube which contained methanol and 0.01 % butylated hydroxytoluene (Sigma) as an antioxidant. Lipids were extracted by the method described by Bligh and Dyer [15]. Then the lipid samples were spotted on thin-layer chromatography (TLC) silica plates (Kieselgel 60, 0.22 mm, Merck) and developed as described by Mahadevappa et al. [16]. Standards of sphingomyelin (Sigma) were run along with the samples. Lipid bands were visualized under ultraviolet light after spraying with a 0.5 % solution of 3′7′-dichlorofluorescein in absolute methanol. The gel bands corresponding to the sphingomyelin were scraped off the plate and transferred into screw tubes containing pentadecanoic acid (Sigma–Aldrich, St. Louis, MO) as an internal standard. Sphingomyelin fatty acids were then transmethylated and subsequently analyzed by means of gas–liquid chromatography. A Hewlett-Packard 5890 Series II system, equipped with a double flame ionization detector and Agilent CP-Sil 88 capillary column (100 m, internal diameter of 0.25 mm), was used. The sphingomyelin content is presented as the sum of individual fatty acid residues.

### Ceramide Content

A small (50 μl) volume of the chloroform phase, containing the lipids extract was transferred to a fresh tube which contained an internal standard (C17-sphingosine, Avanti Polar Lipids, UK). Ceramide (Cer) present in the organic phase was hydrolyzed in 1 M KOH in 90 % methanol at 90 °C for 60 min. This digestion procedure does not convert complex sphingolipids, such as SM, galactosylceramide, or glucosylceramide, into free sphingoid bases [17]. The content of free sphingosine, liberated from Cer was next analyzed by means of HPLC (Varian Inc. OmniSpher 5, 4.6 × 150 mm). The calibration curve was prepared using *N*-palmitoylsphingosine (Avanti Polar Lipids, UK) as a standard. The chloroform extract used for the analysis of Cer level also contains small amounts of free sphingoid bases. Therefore, the content of Cer was corrected for the level of free sphingosine determined in the same sample.

### Sphingosine, Sphinganine and Sphingosine-1-Phosphate Content

The ceramide derivatives were measured according to the method, described by Min et al. [18]. Prior to sample homogenization and ultrasonication, internal standards (C17-sphingosine and C17-S1P, Avanti Polar Lipids, Alabaster, AL) were added. The sphingoid bases were converted to their *o*-phthalaldehyde derivatives and analyzed on a HPLC system (ProStar, Varian, Inc., Palo Alto, CA) equipped with a fluorescence detector and C18 reversed-phase column (Varian, Inc., OmniSpher 5, 4.6 × 150 mm).

### The Activity of SPT Neutral SMase and Acid SMase

The protein content was measured in all homogenates prior to enzymatic analysis with the BSA protein assay kit (Sigma–Aldrich, St. Louis, MO). As a standard, bovine serum albumin (fatty acid free, Sigma–Aldrich, St. Louis, MO) was used.

The activity of SPT, neutral and acid isoforms of sphingomyelinase was determined accordingly [11, 19], with the use of radiolabeled substrate (Perkin-Elmer Life Sciences, Waltham, MA). The product of reaction ^14^C-choline phosphate or ^3^H-L-serine was extracted with CHCl_3_/methanol (2, 1, v/v), transferred to scintillation vials, and counted using a Packard TRI-CARB 1900 TR scintillation counter.

### The Activity of Alkaline (alCDase) Ceramidase and Neutral (nCDase) Ceramidase

The activity of neutral CDase and alkaline CDase was measured by the method described by Nikolova-Karakashian et al. [20]. The activity was determined with the use of radiolabeled [*N*-palmitoyl-1-^14^C]-sphingosine (Moravek Biochemicals, Brea, CA) as a substrate. Unreacted ceramide and liberated 1-^14^C-palmitate were separated with the basic Dole solution (isopropanol/heptane/1 N NaOH, 40, 10, 1, v/v/v). Radioactivity of the 1-^14^C-palmitate was measured by scintillation counting.

### THR Expression

The protein expression of THRα, β-actin (50 μg) was determined in muscle homogenates. The routine Western blotting technique was used to detect the protein content. Briefly, the total protein content in each sample was determined by BCA (bicinchoninic acid) method. Then, the proteins in each sample were separated using 10 % SDS–polyacrylamide gel electrophoresis and transferred to the nitrocellulose membrane. Equal protein concentrations were loaded in each lane as confirmed by Ponceau staining the blot membrane. In the next step, membranes were immunoblotted with selected primary antibodies (THRα, β-actin (Abcam, EU). Quantification of the selected protein content was achieved by densitometry (OD-Optical Density; Biorad, Poland). The THRα expression was related to β-actin and then to the control that was set to 100 %.

### Plasma FFA and Triiodothyronine (T_3_) Concentration

To measure the content of plasma FFA, lipids were extracted from the plasma samples and subsequently the fraction of FFA was isolated by means of TLC (Merck, Germany). The gel bands, corresponding to the FFA standard, were scraped off the plates and transferred into fresh tubes. FFAs were then transmethylated with BF_3_/methanol and the content of their methyl esters was determined by means of gas–liquid chromatography (GLC) [21]. The total content of plasma FFA was estimated as the sum of the particular fatty acid species and was expressed in nanomoles per milliliter of the plasma. T_3_ concentration was measured in serum, with a commercially available kit, according to manufacture instruction (Rat Tri-iodothyronine, T_3_ ELISA kit, EIAab).

### Statistical Analysis

Data are presented as means ± SE. Statistical significance was assessed using two-way ANOVA followed by the Newman–Keuls post hoc test. Differences were considered significant at *p* < 0.05.

## Results

### Effects of T_3_ Treatment on the Plasma T_3_ Concentration and Muscle THR Expression

**Fig. 1:**
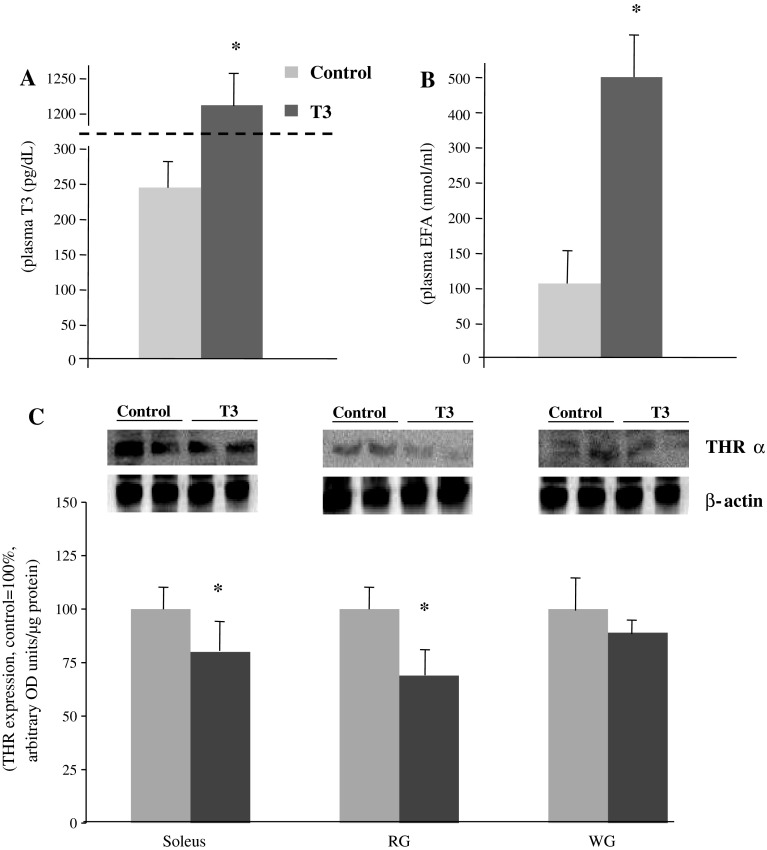
Effects of T_3_ treatment (10 days) on the concentration of plasma T_3_ (**a**), FFA (**b**) and THRα expression (**c**) (Western blot) in muscle homogenates. RG, red section of the gastrocnemius; WG, white section of the gastrocnemius; T_3_, triiodothyronine; THRα, thyroid hormone receptor alpha. Results are based on eight independent preparations for each experimental treatment (mean ± SE). *p < 0.05, control vs treatment (T_3_)

Prior to the sphingolipids examination, we verified the effects of prolonged (10 days) T_3_ i.p. administration on plasma T_3_ concentration and thyroid hormone receptor expression in muscles examined. We found a significant increase in plasma T_3_ (Fig. 1a, +5.0-fold, *p* < 0.05), which resulted in downregulation of the THR expression (Fig. 1c, −21 %, −25 %, *p* < 0.05 and −11 %, *p* > 0,05). Furthermore, plasma FFA content was significantly increased along with T_3_ administration (Fig. 1b, +4.8-fold, *p* < 0.05).

### Effects of T_3_ Treatment on the Sphingolipids Content

**Fig. 2:**
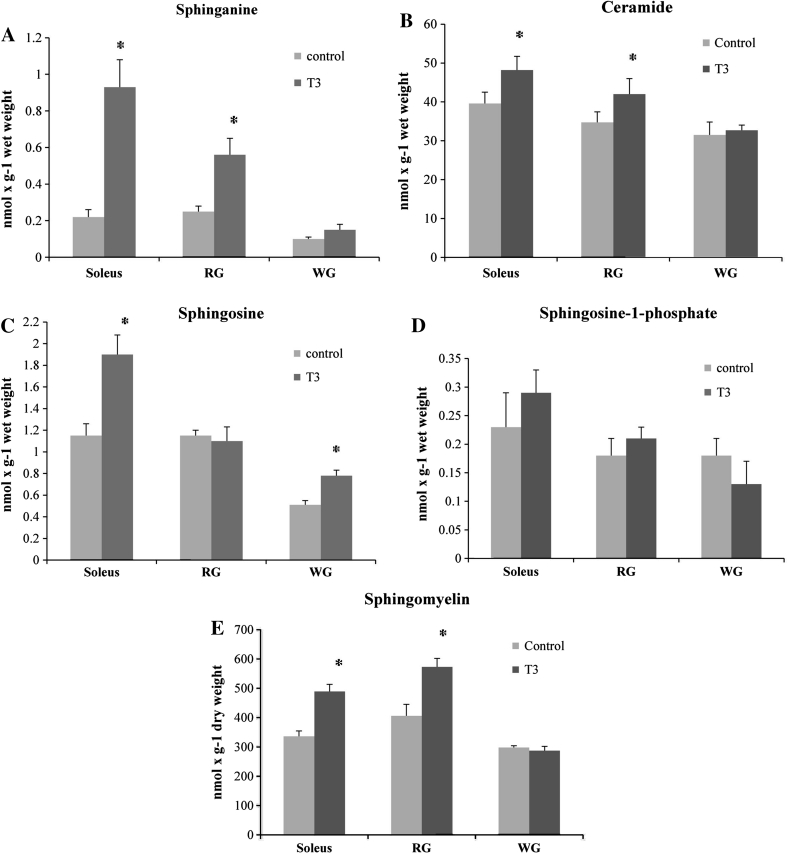
Effects of in vivo T_3_ administration (10 days) on the content of: sphinganine (**a**), ceramide (**b**), sphingosine (**c**), sphingosine-1-phosphate (**d**) and sphingomyelin (**e**) in rat skeletal muscles. RG, red section of the gastrocnemius; WG, white section of the gastrocnemius; T_3_, triiodothyronine. Results are based on eight independent preparations for each experimental treatment (mean ± SE). *p < 0.05, control vs treatment (T_3_)

Prolonged high levels of serum T_3_ influenced sphingolipids metabolism in skeletal muscles. We noticed an increased content of sphinganine (an important substrate for de novo synthesis of ceramide), that was significantly higher in more oxidative muscles of hyperthroids rats, namely in soleus and in the red section of gastrocnemius (Fig. 2a, 4.2-fold and 2.2-fold, *p* < 0.05, respectively). Subsequently, we observed significant elevation of the content of ceramide in soleus and red gastrocnemius (Fig. 2b, +22 and +21 %, *p* < 0.05, respectively), accompanied by a significant increase in sphingosine content (Fig. 2c, +65 and +53 %, *p* < 0.05, respectively). In contrast, hyperthyroidism had no significant effects on sphingosine-1-phosphate content in the muscles studied (Fig. 2d), although a trend towards an increase in S1P was noticed in soleus (*p* = 0.064). Hyperthyreosis induced enhancements in the content of sphingomyelin in both soleus and red gastrocnemius (Fig. 2e, +45 % and +41 %, *p* < 0.05, respectively) with modest effects on sphingomyelin concentration in white gastrocnemius.

### Effects of T_3_ Treatment on the Activity of Key Enzymes Involved in Sphingolipid Metabolism

**Fig. 3:**
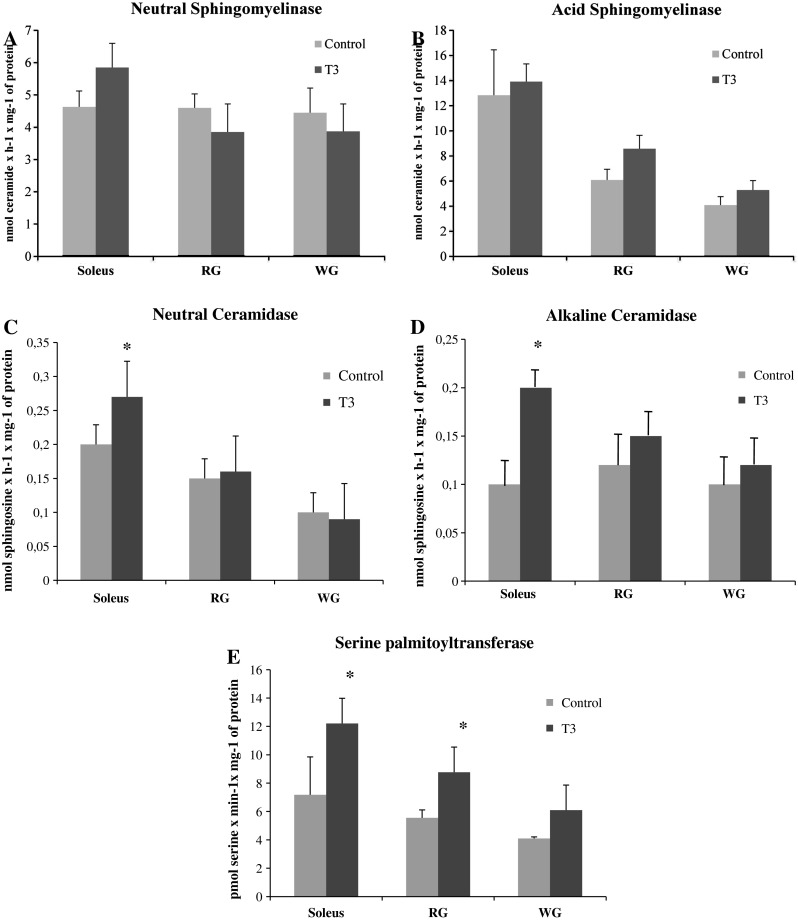
Effects of in vivo T_3_ administration (10 days) on the activity of neutral sphingomyelinase (**a**), acid sphingomyelinase (**b**), neutral ceramidase (**c**), alkaline ceramidase (**d**), serine palmitoyltransferase (**e**) in rat skeletal muscles. RG, red section of the gastrocnemius; WG, white section of the gastrocnemius; T_3_, triiodothyronine. Results are based on eight independent preparations for each experimental treatment (mean ± SE). *p < 0.05, control vs treatment (T_3_)

T_3_ treatment had only a minor effect on the activity of either neutral or acid sphingomyelinases in each muscle studied (Fig. 3a, b). Correspondingly, prolonged T_3_ treatment did not induce significant changes in the activity of neutral ceramidase in red and white gastrocnemius (Fig. 3c, *p* > 0.05), but increased activity in soleus (Fig. 3c, 45 %, *p* < 0.05). Furthermore, hyperthyreosis induced enhancements in the activity of alkaline ceramidase in soleus (Fig. 3d, +110 %, *p* < 0.05), but not in gastrocnemius (Fig. 3d, *p* > 0.05). In hyperthyroid rats the activity of serine palmitoyltransferase was significantly greater in soleus and red than in the respective controls (Fig. 3e, +70 %, +58 %, *p* < 0.05) and only a trend was observed in white gastrocnemius (Fig. 3e, +48 %, *p* = 0.07).

## Discussion

The present study examined the effects of hyperthyroidism on sphingolipid metabolism in three different types of skeletal muscles. We have demonstrated that prolonged, in vivo, T_3_ administration increased the content of both sphinganine, ceramide, and sphingosine in soleus and red gastrocnemius, but not in the white portion of gastrocnemius muscle. Accordingly, in these muscles, the enhancement in sphingolipid content was accompanied by a greater activity of serine palmitoyltransferase (SPT) and, to a lesser extent ceramidases (n-CDase, al-CDase). This indicates that hyperthyroidism accelerates sphingolipid metabolism in a fiber-specific manner, mainly via increased de novo ceramide synthesis.

It is well known, that triiodothyronine (T_3_) exerts pronounced effects on body metabolism, affecting not only energy utilization, but also major cellular signaling pathways [5], including the sphingomyelin pathway [1, 22]. In addition, molecular actions of T_3_ (via activation of its receptors) in skeletal muscles, are fiber specific [4, 23]. Recent data point to a greater responsiveness for T_3_ treatment in soleus muscle (slow-twitch oxidative fibers) than in plantaris (fast-twitch oxidative-glycolytic mixed muscle). This greater response was mainly due to the increased peroxisome proliferator activated receptor coactivator 1α (PGC-1α) expression, followed by increased size and number of mitochondria in soleus but not in plantaris muscle [3, 24]. The aforementioned study probably explains T_3_-induced increased rates of fatty acid metabolism in more oxidative fibers comparing to glycolytic muscles. Furthermore, it is well established, that triiodothyronine can regulate muscle metabolism via multiple molecular mechanism, including direct AMPK activation [25] or through elevated calcium levels and CaMKKβ activation [26]. Hyperthyroidism induced AMPK activation substantially increased substrate metabolism in heart [27] as well as in skeletal muscle [28]. However, there are only a few studies addressing the influence of AMPK activation on the activity of enzymes involved in ceramide metabolism [29, 30] and it is still unclear whether increased rates of palmitate metabolism are directly related to the rates of ceramide metabolism. Indirect evidence is provided by studies showing increased ceramide content in skeletal muscles after exercise during which AMPK is activated [11, 12]. Presumably, greater rates of fatty acid utilization, along with a higher content of neutral lipids (such as triacylglycerols) [31] may result in faster rates of ceramide metabolism in more oxidative muscles. Accordingly, in the present study as well as in previous reports [32], higher concentrations of ceramide and other sphingolipids in these muscles were noticed. Also, along with greater content of ceramide, we observed increased activity of the key enzymes involved in sphigolipids metabolism in more oxidative fibers. Furthermore, it seems that different skeletal muscles do not contain the same number of thyroid hormone receptors, resulting in a differential sensitivity of muscle types to the hormone [5]. Taken together these results suggest that T_3_ influences the skeletal muscles sphingolipids profile in a fiber specific manner. However, one can question that increased ceramide metabolism is solely due to direct T_3_ actions in skeletal muscles since, in vivo, key enzymes involved in sphingolipid metabolism are regulated by many physiological and environmental stimuli [11]. It is therefore possible, that T_3_ induced changes in ceramide metabolism are secondary, and are due to the increased availability of serum fatty acids, provoked by triiodothyronine-induced adipose tissue lipolysis. Probably, this may be the case in our study, since we observed increased serum FFA followed by greater activity of serine palmitoyltransferase (a key enzyme responsible for de novo generation of ceramide) [33]. It is well known, that the activity of SPT is directly stimulated by the presence of long chain fatty acids (e.g. palmitate, a major representative of serum FFA) [34] and some studies show that de novo ceramide synthesis can be driven exclusively by increased availability of fatty acids [35, 36]. Further, indirect evidence, for increased de novo ceramide generation can be drawn from our observations of the greater content in sphinganine than sphingosine, especially since sphinganine is considered to be a key intermediate substrate in the de novo ceramide synthesis pathway and sphingosine is one of the major ceramide degradation products [8]. It is probable that the sphingolipid changes were closely related to increased activity of either SPT-1 and ceramidases (neutral and alkaline CDase), but interestingly the activity of sphingosine kinase (SPHK) must have remained quite stable, since sphingosine-1 phosphate levels did not change significantly. Beyond this correlation, several studies have provided evidence for relatively low expression and activity of SPHK in skeletal muscles [37]. In contrast, it is also plausible that increased activity of sphingomyelinase may contribute to ceramide accumulation, through the enhanced hydrolysis of sphingomyelin. Recently increased abundance/activity of either neutral or acid sphingomyelinase that generated increased ceramide levels, has been reported in the adipose tissue of ob/ob and high fat diet induced obese mice [38] and rats [39], but in the present study we did not observe significant T_3_-induced changes in the activity of sphingomyelinase.

To summarize, we have shown that hyperthyroidism increases sphingolipid metabolism in skeletal muscles in a fiber specific manner, exclusively in more oxidative muscles. T_3_ induced accumulation of ceramide in more oxidative muscles was further related to de novo synthesis rather than to the hydrolysis of sphingomyelin.

## Abbreviations

T_3_: Triiodothyronine
FFA: Free fatty acids
SPA: Sphinganine
SPH: Sphingosine
S-1-P: Sphingosine-1-phosphate
CER: Ceramide
SM: Sphingomyelin
aSM-ase: Acid sphingomyelinase
nSM-ase: Neutral sphingomyelinase
alCDase: Alkaline ceramidase
nCDase: Neutral ceramidase
SPT: Serine palmitoyltransferase
THR: Thyroid hormone receptor
